# Bone Niches, Hematopoietic Stem Cells, and Vessel Formation

**DOI:** 10.3390/ijms18010151

**Published:** 2017-01-13

**Authors:** Roberto Tamma, Domenico Ribatti

**Affiliations:** 1 Department of Basic Medical Sciences, Neurosciences and Sensory Organs, Section of Human Anatomy and Histology, University of Bari Medical School, 70124 Bari, Italy; roberto.tamma@uniba.it; 2 National Cancer Institute Giovanni Paolo II, 70124 Bari, Italy

**Keywords:** angiogenesis, bone marrow, endosteal niche, hematopoietic stem cells, vascular niche, vasculogenesis

## Abstract

Bone marrow (BM) is a source of hematopoietic stem cells (HSCs). HSCs are localized in both the endosteum, in the so-called endosteal niche, and close to thin-walled and fenestrated sinusoidal vessel in the center of BM, in the so-called vascular niche. HSCs give rise to all types of mature blood cells through a process finely controlled by numerous signals emerging from the bone marrow niches where HSCs reside. This review will focus on the description of the role of BM niches in the control of the fate of HSCs and will also highlight the role of the BM niches in the regulation of vasculogenesis and angiogenesis. Moreover, alterations of the signals in niche microenvironment are involved in many aspects of tumor progression and vascularization and further knowledge could provide the basis for the development of new therapeutic strategies.

## 1. Introduction

Many tissues keep their intrinsic property of regeneration in postnatal life, a process crucial for lifelong maintenance of organ function. This is possible through the presence of particular cellular elements named stem cells, an important source of new cells. Stem cells have not completed their differentiation path and are characterized by two properties: self-renewal and unlimited potency. Self-renewal of a cell is its ability to go through numerous cycles of division while maintaining the undifferentiated state. Unlimited potency means the capacity to differentiate into any mature cell type, characteristic of embryonic stem cells. In postnatal life, stem cells are multipotent, which means they are able to differentiate into multiple, but limited cell types. An example of stem cells is hematopoietic stem cells (HSCs), which give rise to all the blood cells [1,2]. HSCs are able to preserve genomic integrity, maintain functional capacity, and proliferate and differentiate [3].

HSCs are not spread throughout the body but are organized in particular microenvironments called niches localized in the bone marrow (BM) cavity that include the endosteal and the vascular niches [4,5,6]. Both are fundamental source of instructive signals that maintain and regulate the activity of HSCs throughout life [7,8]. Moreover, numerous chemical mediators and the extracellular matrix play an important role in the regulation of HSC fate.

This review article will be focused in the first two sections on recent findings concerning the structure and characteristics of cells and the main signals supporting the hematopoietic process, including motility, transendothelial migration, and hematopoietic differentiation. Moreover, the bone marrow is also considered a source of endothelial precursor cells able that participate in the growth of blood vessels during postnatal vasculogenesis as well as in the angiogenic process. In the last two sections, we will elucidate the role of the bone marrow niches in the release of endothelial precursors into the circulation and their recruitment to the angiogenic sites during both physiological and pathological processes.

## 2. Endosteal Niche

**Figure 1:**
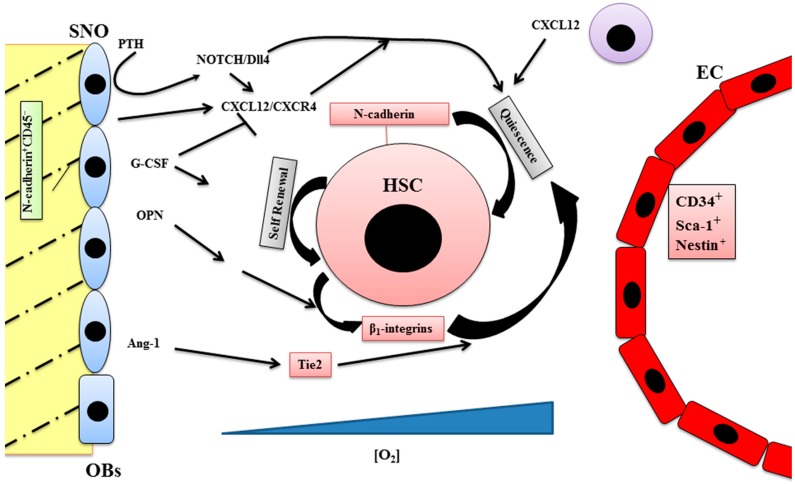
The endosteal niche is a complex structure inside which all the components, such as stem cells, progenitor cells, stromal cells, growth factors, and extracellular matrix (ECM) molecules participate in the regulation of hematopoiesis. Spindle-shaped N-cadherin^+^CD45^−^ osteoblastic cells (SNO); osteoblasts (OBs); endothelial cells (EC); hematopoietic stem cells (HSC); granulocyte colony-stimulating factor (G-CSF) ; osteopontin (OPN); parathyroid hormone (PTH); δ like ligand 4 (Dll4); C-X-C chemokine receptor type 4 (CXCR4); stromal cell derived factor-1/C-X-C motif chemokine 12 (CXCL12); angiopoietin receptor-2 (Tie2); angiopoietin-1 (Ang-1); Notch/translocation−associated Notch homologue (NOTCH). Blue triangle: oxygen gradient.

The endosteal niche is localized in the internal bone shell surface [9], in particular close to the endocortical and trabecular surfaces [4] (Figure 1). The endosteum is a histological structure that interposes between bone and bone marrow; it principally includes bone-forming osteoblasts and bone-resorbing osteoclasts, and other cells including fibroblasts, macrophages, endothelial cells, and adipocytes are localized near the endosteum. There is significant evidence that osteoblasts influence HSC maturation [10].

Two types of osteoblastic cells are present in the endosteum, the actively bone-producing osteoblasts and the quiescent bone-lining cells, a type of early osteoblast termed spindle-shaped N-cadherin^+^CD45^−^ osteoblastic cells (SNO) [11]. SNO–HSC interactions are critical for the maintenance of stem cell properties. The expression of N-cadherin in HSCs contributes in maintaining them in quiescence and its depletion leads to an impairment of HSCs bond to the endosteal surface [12,13,14]. Although the importance of homophilic N-cadherin-mediated binding in SNO–HSC interaction emerges from these data, N-cadherin in SNO is not required for maintenance of HSCs [15]. This discrepancy could be explained considering the involvement of other types of cadherin as compensatory mechanisms when the loss of N-cadherin in SNO occurs during embryonic development.

Other osteoblast and HSCs surface molecules are involved in the regulation of endosteal HSCs’ fate. It has been demonstrated that HSCs that adhere to osteoblasts on the bone surface express angiopoietin receptor-2 (Tie2), while the lining cells, in adult BM, produce angiopoietin-1 (Ang-1). The activation of Tie-2 by Ang-1 in vitro induces the adhesion of the two types of cells and consequently the immature HSCs phenotype. Moreover, Ang-1 promotes quiescence, enhances the survival of HSCs, and protects them from various stresses [16,17].

The role of the correct differentiation of osteoblasts in maintaining HSCs has also been investigated. Mice defective for Runx2 expression, a transcription factor involved in osteoblast differentiation, showed profound alteration of osteoblast maturation and a total lack of BM throughout the entire skeleton. In the meantime, Runx-2 does not have significant effects on the ontogeny of definitive hematopoietic cells in the yolk sac and liver [18].

When the osteoblasts undergo death, the BM loses its hematopoietic property, which results in the hematopoietic enhancement of extramedullary sites such as the liver and spleen; these experiments underline the role of osteoblasts in BM hematopoiesis. Immature osteoblasts, with high Runx2 and low osteocalcin expression, but expressing the adhesion molecule CD166, are responsible of hematopoiesis-enhancing activity, even if functional studies using conditional knockout (KO) mice revealed the scant contribution of osteoblast to HSC.

In mice, in long bone-HSC transplantation studies, the majority of the cells first colonize the endosteal epiphyses’ surfaces, forming clusters and secondarily formed new clusters in the more central regions of the BM [4,19]. Moreover, in areas where new trabecular bone is formed and its surface enriched with osteoblasts, the number of HSCs increased [14].

Also, the oxygen gradient guides the localization of HSCs in the endosteal region. HSCs show a selective redistribution within the endosteal region, whereas lineage-committed and mature cells were selectively redistributed towards the central marrow region so that hematopoietic differentiation seems to proceed radially toward the longitudinal axis of the marrow [20]. This could be related to the oxygen gradient into the BM cavity, where HSCs are predominantly located at the lowest side of the gradient because the regionally defined hypoxia plays a fundamental role in regulating stem cell function [21]. Reactive oxygen species (ROS) may be involved in this mechanism since a correspondence between the increased cycling and apoptosis of HSCs and the increased levels of ROS has been found, an effect caused by the administration of the antioxidants [22].

Under the influence of parathyroid hormone (PTH) or the locally produced PTH-related protein (PTHrP), the number of osteoblasts increases, resulting in the expansion of HSCs mediated by Jagged1 (JAG1)-dependent activation of Notch1. HSC quiescence and retention in the marrow niche is facilitated by the interaction between Notch1 and JAG1 or δ-like ligand 4 (Dll4) expressed by niche cells [23]. In conditional mutant mice defective for Jagged1 and Notch1 in the BM compartment, HSCs undergo self-renewal and differentiation through other Notch receptors and/or ligands replacing Jagged1 and Notch1 [24]

A relationship exists between Notch and stromal cell derived factor-1 (CXCL12), a small chemokine belonging to the CXC subfamily with a strong chemoattractant property, and its receptor, C-X-C chemokine receptor type 4 (CXCR4) In mesenchymal stem cells migration and function are regulated by the Notch/Dll4 pathway [25], as well as the expression of CXCR4 in endothelial cells [26]. Immature osteoblasts in the endosteal niche express higher levels of CXCL12 and support HSC maintenance [27]. There is evidence that CXCL12 is constitutively produced by BM stromal cells [28] and regulates HSC mobilization in the BM as well as cell adhesion, survival, and cell-cycle status [29]. Finally, CXCL12 expression is negatively modulated by granulocyte-colony stimulating factor (G-CSF) treatment and CXCR4 on HSC may be inactivated during G-CSF treatment [30].

Stem cell factor (SCF) stimulates proliferation and differentiation of HSCs in vitro [30], and SCF receptor, c-kit, is highly expressed on quiescent HSCs in adult BM [31,32,33]. Its role in maintaining HSCs in BM has been investigated in mice, in which the partial loss of c-kit is associated with the reduction of HSCs.

The role of cell–extracellular matrix (ECM) interaction in the control of HSCs has been extensively investigated.

Osteopontin (OPN), involved in the regulation of cell–cell and cell–ECM interactions [34], is expressed in bone and extra-bone sites [35]. Osteoblasts negatively regulate stem cell pool size [36] by the expression of OPN on their membrane [37]. In OPN null mice, OPN promotes HSC quiescence and plays a chemoattractant role for HSCs toward the endosteal region [38]. This process involves β_1_-integrins, expressed in HSCs, and treatment with antibodies anti-β1 integrins prevents the homing of hematopoietic progenitors to the adult BM and spleen [39,40]. Moreover, both α4β1 and α9β1integrins expressed by HSCs are implicated in niche retention in the endosteal region [38]. In fact, α9β1/α4β1 antagonists contribute to rapid and transient HSCs mobilization during transplant of mobilized peripheral blood HSCs into patients undergoing treatment for blood diseases [41].

Null mice for thrombopoietin receptor (THPO) show thrombocytopenia and reduction in progenitor cells of several hematopoietic lineages [42], and THPO and its receptor are expressed on HSCs and the osteoblast surface in the endosteal niche.

Although in the endosteal niche the regulatory signals released from neighboring cells in the form of bound or secreted molecules and physical signals such as oxygen tension are crucial in the control of HSCs quiescence and activation, the soluble factors and signaling pathways present in the vascular niche are important in HSC self-renewal and maintenance.

## 3. Vascular Niche

The blood vessels of the BM not only constitute the wall that separates the hematopoietic compartment from the peripheral circulation but are able to regulate hematopoiesis as well as stem cell mobilization and homing.

**Figure 2:**
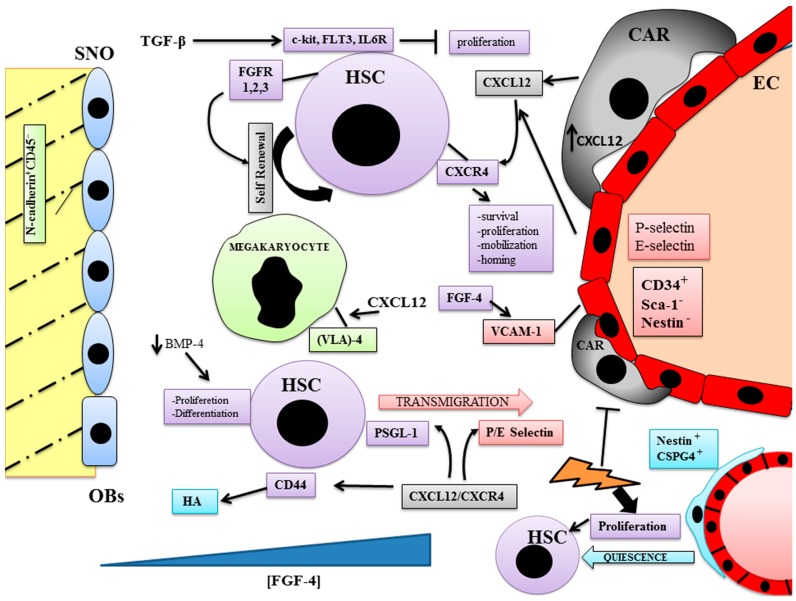
In the vascular niche hematopoiesis occurs in the extravascular spaces between the sinuses. The medullary vascular sinuses are lined with endothelial cells and surrounded by adventitial cells, called CXCL12-abundant reticular (CAR) cells. The closeness between sinusoidal endothelial cells and HSCs is very important for their maturation and so for the hematopoietic process. An arteriolar niche has been found where quiescent HSCs are associated with a cell population different from CAR, named peri-arteriolar nestin cells. These cells express chondroitin sulfate proteoglycan-4 (CSPG4), show chemoresistance after genotoxix injury, and activate HSC proliferation. CXCL12-abundant reticular cells (CAR); fibroblast growth factor receptors (FGFRs); transforming growth factor-β (TGF-β); bone morphogenetic protein (BMP); hyaluronic acid (HA); glycoprotein ligand-1 (PSGL-1); very late antigen (VLA-4); chondroitin sulfate proteoglycan-4 (CSPG4); fibroblast growth factor-4 (FGF-4); P/E selectin type P and E (P/E selectin); vascular cell adhesion molecule 1 (VCAM-1); FMS like tyrosine kinase 3 (FLT3); interleukin 6 receptor (IL6R). Blue triangle: FGF-4 gradient; lightning sign: genotoxic stimulation.

BM can be divided into stromal and parenchymal compartments. The parenchyma includes the different immature and mature blood cell precursors [43] in direct contact with the sinusoids CD34^+^ nestin and Sca-1^−^, while endosteal endothelial cells express all these three markers [44]. Sinusoids are radially distributed around the draining central sinus [45], and hematopoiesis occurs in the extravascular spaces between the sinuses [46]. The medullary vascular sinuses are lined with endothelial cells and surrounded by adventitial cells, a small population of cells with long processes, expressing high amounts of CXCL12, called CXCL12-abundant reticular (CAR) cells. CAR cells are a crucial component of niches for HSCs [47]. Moreover, the stromal cells, including mesenchymal stem cells (MSCs), form a reticular network that supports HSC formation [48] (Figure 2).

The closeness between sinusoidal endothelial cells and HSCs is very important for their maturation [48]. The link between hematopoiesis and the THPO/cMpl system is well known, and in particular its role in promoting the proliferation of megakaryocytic progenitors [46]. Despite the absence of the THPO/cMpl system, megakaryocyte maturation and platelet production may take place if megakaryocyte progenitors are close to BM sinusoids [49]. In THPO and cMpl KO mice where thrombopoiesis is compromised, CXCL12 and fibroblast growth factor-4 (FGF-4) restore the normal platelet through a mechanism that induces the expression of adhesion molecules, including very late antigen (VLA)-4 on megakaryocytes and VCAM-1 on endothelial cells [50,51]. This underlines how the cell–cell interaction between sinusoid endothelial cells and megakaryocytes is fundamental in the control of maturation of the latter, and emphasizes the importance of the different roles played by the endosteal and vascular niches. FGF may be considered an important factor in mediating the crosstalk between the vascular and the endosteal niche [52]. It is thought that FGF forms a gradient between the two BM niches, important in the recruitment of HSCs and their progenitors to the vascular niche, where high expression of FGF receptors has been found [53].

Transforming growth factor-β (TGF-β) is a negative, reversible regulator of HSCs proliferation in vitro [54] and is thought to be a key regulator of HSC quiescence in vivo. TGF-β regulates the expression of many cytokine receptors as c-kit, FLT3, and IL6R, all implicated in the control of HSC biology and in the upregulation of cyclin-dependent kinase inhibitors such as p21 and p57 [55,56]. Bone morphogenetic proteins (BMPs) are growth factors belonging to the TGF-β superfamily [57]. High concentrations of BMP-2 and -7 inhibit HSC proliferation, whereas BMP-4 at high concentrations has an effect on stem cell survival; a lower concentration stimulates stem cell proliferation and differentiation [58].

WNT5a shows hematopoietic properties in both adult and fetal human BM and is expressed by Lin^−^CD34^+^ HSCs progenitors [40]. Moreover, WNT3a stimulates murine HSCs self-renewal [59]. Data about the intracellular mechanisms by which WNTs exert their effect on HSCs are controversial due to the opposite effects of WNTs in vitro and in vivo [60]. Notch signaling is required for WNTs’ action in HSC maintenance, but not for survival or replication in vitro [61]. WNT3a regulates the expression of Notch target genes and inhibition of the WNT signaling affects HSC fate. It is believed that the two pathways belong to a network of regulatory circuits controlling the HSC pool [62].

Both the endothelial and the stromal cells express CXCL12, while HSCs express CXCR4; it is known that the CXCL12/CXCR4 system is crucial for cell survival, proliferation, and regulating the mobilization and the homing of HSCs. In response to CXCL12/CXCR4 signaling, endothelial cells express P-selectin and E-selectin [63] and HSCs express glycoprotein ligand-1 (PSGL-1), which interacts with both the endothelial selectins regulating the transmigration of HSCs [63]. Moreover, HSCs and progenitor cells express the smallest isoform of CD44, a cell-surface glycoprotein that is involved in cell migration for a variety of normal and malignant cells [64]. Neutralizing anti-CD44 antibodies impaired homing of mouse hematopoietic progenitors to the BM and spleen, whereas in CD44 knock-out mice a reduced myeloid progenitor cell migration from the BM to blood circulation occurs [65,66]. The role of CD44 in hematopoiesis is contrasting because the antibodies anti CD44 promoted or inhibited this process [67,68].

Hyaluronic acid (HA) is synthesized by primitive hematopoietic cells and participates in their migration to the endosteal niche after transplantation [69]. CXCL12 stimulates the adhesion of progenitor cells to HA [70]. HA is expressed on the human BM sinusoidal endothelium and endosteum, where CXCL12 is highly expressed; in this context, CD44, HA, and CXCL12 regulate the transendothelial migration of HSCs and progenitors and their final anchorage within specific niches of the BM [59].

Another vessel-associated niche involved in maintaining the balance of HSCs between proliferation and dormancy has been identified in the bone marrow, consisting of a type of arterioles structurally distinct from the sinusoid, as highlighted in a recent study [71]. In this arteriolar niche it has individuated a population named peri-arteriolar nestin and chondroitin sulfate proteoglycan-4 (CSPG4) expressing cells, distinctive from the CAR cells. Genotoxic experiments revealed the chemoresistance of the nestin perivascular cells compared to CAR, which were instead subject to destruction, allowing us to postulate that the arteriolar niche is crucial in orchestrating hematopoietic and stromal regeneration after injury.

These data showed the important role of the vascular niche in the regulation of hematopoiesis and in supporting the self-renewal of HSCs as well as the crosstalk existing between the two BM niches being involved in the regulation of HSCs fate.

## 4. Bone Marrow Niches and Vessel Formation

BM, in addition to its crucial role in the hematopoietic process, is also involved in the process of vasculogenesis. Vasculogenesis, originally described as embryonic blood vessel formation from the endothelial cell precursor, is also referred to as the formation of new vessels from endothelial precursor cells (EPCs) in postnatal life [72]. Vasculogenesis leads to the formation of the first major intra-embryonic blood vessels, such as the dorsal aorta and the cardinal veins, and to the formation of the primary vascular plexus in the yolk sac. With the onset of embryonic circulation, these primary vessels have to be remodeled into arteries and veins in order to develop a functional vascular loop. Remodeling of the primary vascular plexus into a more mature vascular system is thought to occur by a process termed angiogenesis. The term angiogenesis, applied to the formation of capillaries from pre-existing vessels, i.e., capillaries and postcapillary venules, is based on endothelial sprouting microvascular growth.

BM-derived endothelial cells (BMDECs) are involved in indirectly promoting [73] vascular growth through the expression of angiogenic factors at the site where the neovascularization occurs [74]. CXCR4 is highly expressed by BMDECs and is involved in their mobilization and homing [75,76]. CXCL12 expression is also directly regulated by vascular endothelial growth factor (VEGF). The role of VEGF in BMDECs’ recruitment has been studied, inducing its expression in a transgenic system without other stimuli such as hypoxia. VEGF stimulates the expression of CXCL12 in perivascular cells and the latter attracts CXCR4^+^ circulating cells. Blocking VEGF receptor-1 (VEGFR-1) reduces the number of recruited perivascular cells in tumors [77], suggesting that the effect of VEGF could involve VEGFR-1. Overall, these data confirm that VEGF is a pro-angiogenic growth factor that stimulates BMDECs through the recruitment of perivascular cells and the activation of VEGFR-1.

A mechanism proposed for the release of BMDECs from BM involves matrix metalloproteinase-9 (MMP-9). In hypoxic conditions, VEGF-A and CXCL12 are upregulated and induce the release of activated MMP-9 within the BM cell niches, which activates soluble kit-ligand, resulting in the release of BMDECs into the peripheral blood [78]. Studies on mice mutant for β3 phosphorylation sites (DiYF) showed the presence of a high number of circulating CXCR4^+^BMDECs as well as the loss of the ability of BMDECs derived from DiYF mice to transmigrate through the endothelial monolayer [79], suggesting that the presence of complete β3 integrin activity is crucial for the recruitment of BMDECs from the circulation into target tissues.

EPCs are bone-marrow-derived cells, functionally and phenotypically distinct from mature endothelial cells, with the ability to differentiate in endothelial cells in vitro and contribute to new blood vessels formation [80,81]. EPCs directly form new vessels and are a rich source of pro-angiogenic factors [82]. Primitive EPCs expressing CD133, CD34, and VEGFR-2 differentiate in a mature form that loses CD133 expression [50]. VEGF is a strong inducer of EPC mobilization [72] both in vitro and in vivo, and mobilization of EPCs into the peripheral circulation is increased after human recombinant VEGF administration in vivo [83]. Also, GM-CSF participates in EPCs’ mobilization from BM [84], and osteoblast progenitors respond to hypoxia or insulin-like growth factor-1 (IGF-1), augmenting hypoxia inducible factor (HIF) signaling that results in HSC niche expansion associated with selective expansion of the erythroid lineage [85]. The effect of erythroid lineage seems to be directly related to erythropoietin (EPO) expression in osteoblasts [86]. EPO is a key molecule in the process of vascular repair and neoangiogenesis and affects EPCs activity by increasing mobility and enhancing their ability to form tubes [87,88,89].

BM appears to be an important source for mobilized progenitor/stem cells involved not only in the vasculogenesis process but also responsible for new vessel formation through the angiogenesis process. New blood vessel formation in adults contributes to both physiological and pathological processes, including tissue ischemia, repair and regeneration, atherosclerosis, tumor growth, and metastasis [90]. Angiogenesis is crucial for the maintenance and migration of HSCs, their progeny, and supportive cells to and from the BM niche.

New vessels formed through angiogenesis during endochondral ossification are a source of perivascular osteoprogenitor cells and osteoclasts, important for modeling and remodeling processes that ensure correct skeletal development and growth [91]. Bone cells secrete pro-angiogenic factors such as VEGF, which interacts with VEGFR-expressing cells including the endothelial cells, chondrocytes, osteoblasts, and osteoclasts. In the same way, endothelial cells release factors that regulate chondrocytes and cells of the osteoblast lineage [92]. During angiogenesis, which occurs in endochondral ossification, endothelial cells of the advancing capillaries directly and indirectly influence the matrix resorption by producing proteases and regulatory molecules and by recruiting osteoclast precursors from the circulation. Moreover, endothelial cells release VEGF in response to the secretion of HIF1-α by hypoxic chondrocytes [93], and produce BMP-2 and BMP-4, stimulators of osteoblast differentiation [94].

Endothelin-1 (ET-1), expressed by endothelial cells, regulates angiogenesis directly by promoting endothelial cell migration, proliferation, and differentiation, or indirectly by inducing VEGF production in endothelial cells. The stimulation of ET-1 receptors in osteoblasts induces both their differentiation and the VEGF expression [95].

Osteoclasts are also involved in the angiogenesis process. Some authors have indicated that VEGF constitutes a chemoattractant factor for osteoclast precursors [96], and an autocrine/paracrine action of VEGF in osteoclasts has been found. Osteoclasts express VEGF in response to HIF1α, increased by the RANKL activation during osteoclast differentiation [97]. During bone resorption, the TGFβ1 released from the bone matrix induces VEGF expression in the bone-resorbing compartment, which consequently stimulates endothelial activity and supports angiogenesis.

Also, osteocytes contribute to angiogenesis. It is thought that during bone damage the osteocytes that undergo apoptosis express VEGF [98]. Moreover, it has been found that the pulsatile fluid shear stress stimulation of MLOY4 osteocytes induces the secretion of VEGF [99].

Notch/Dll4 signaling is involved in angiogenesis in adult long bones. Arteries express Dll4 and JAG1, the latter also in perivascular osteoprogenitor cells. The role of the Notch/Dll4 system is to stimulate vessel growth and endothelial proliferation by regulating VEGFR expression. Moreover, mice with an impaired Notch/Dll4 pathway showed a reduction in long bone development and an increased number of immature osteoblasts [100].

Another local bone factor differentially expressed and secreted by BM sinusoidal endothelial cells within the vascular niche is pleiotrophin (PTN), a heparin-binding growth factor [101] involved in angiogenesis in vivo and in vitro [102]. IPTN exerts chemotaxis of pro-angiogenic early EPCs in a NOS-dependent manner [103], and stimulates both osteoblast proliferation and bone matrix deposition [104].

## 5. Conclusions

Endothelial cells and EPCs establish a peculiar microenvironment in the bone vascular niche, where upregulation of angiogenic factors and inflammatory cytokines promotes tumor growth and invasiveness potential. The identification of the mechanisms by which endothelial cells convey instructive signals to promote tumor growth may allow us to design new therapeutic strategies to treat angiogenesis-dependent tumors.

In the meantime, infiltrating hematopoietic cells from the bone marrow contribute to angiogenesis in a paracrine manner by secreting angiogenic factors or remodeling the extracellular matrix. On the other hand, HSCs reside in stable, complex microenvironments within bone, control the self-renewal of the microvasculature, and are recruited to the local microenvironment in angiogenesis, especially during tumor angiogenesis. It is unknown whether HSCs that reside in the vascular and endosteal niches have similar functional properties. Moreover, the mechanisms that govern HSC–tumor cell cross-talk with endothelial cells provide promising new directions for future study.
